# Baseplate fixation in reverse shoulder arthroplasty: influence of intraoperative guidance on postoperative outcomes

**DOI:** 10.1016/j.xrrt.2025.100615

**Published:** 2025-11-19

**Authors:** Andrea Estfeller, Alp Paksoy, Ayham Jaber, Yamac Akgün, Doruk Akgün

**Affiliations:** a Medical University of Vienna, Vienna, Austria; b Department of Shoulder and Elbow Surgery, Center for Musculoskeletal Surgery, Charité, Berlin, Germany; c Steadman Philippon Research Institute, Vail, CO, USA; d The Steadman Clinic, Vail, CO, USA; e Department of Pathology and Laboratory Medicine, University of Miami Miller School of Medicine, Miami, FL, USA

**Keywords:** Reverse shoulder arthroplasty, Postoperative outcomes, Glenoid baseplate fixation, Intraoperative computer-assisted navigation, Patient-specific instrumentation, Clinical outcomes, Failure rates

## Abstract

In reverse total shoulder arthroplasty, precise positioning of the glenoid baseplate is essential for optimizing biomechanics and implant longevity. This narrative review introduces modern guidance modalities (patient-specific instrumentation, computer-assisted navigation, and augmented reality intraoperative navigation) and compares available literature on implant alignment accuracy, patient-reported outcomes, and complication rates. Although patient-specific instrumentation and computer-assisted navigation consistently improve alignment with preoperative plans and reduce errors in guide pin and screw placement, short-term measures of strength, mobility, and patient-reported outcomes are similar to those achieved with conventional freehand methods. However, guided approaches demonstrate a reduction in the risk of complications in the short term. This suggests that the primary clinical advantage of these modern techniques is reducing complications and potentially improving long-term implant outcomes.

Over the past decade, the use of reverse total shoulder arthroplasty (rTSA) has increased sharply, driven by an expansion of its applications, ranging from rotator cuff tear arthropathy to complex revision procedures.^1^^,^^8^^,^^22^ Accurate glenoid baseplate positioning is central to the biomechanical success and longevity of rTSA. Even small deviations in version, inclination, or medial-lateral offset resulting in malposition can significantly alter joint loading, accelerate polyethylene wear, lead to scapular notching, and increase the risk of early instability or fixation failure.^8^^,^^37^ A recent retrospective cross-sectional study has shown that glenoid and humeral component malposition is frequent in patients requiring revision arthroplasty.^32^ Furthermore, it has been demonstrated that surgeons malpositioned the glenoid component in 38% of cases, regardless of their expertise.^31^

To enhance positioning accuracy, considerable effort has gone into improving preoperative and intraoperative guidance. Two-dimensional (2D) and three-dimensional (3D) imaging-based planning allow surgeons to visualize patient-specific anatomy and plan for optimal baseplate alignment. However, preoperative planning alone does not assure a perfect execution intraoperatively.^31^^,^^32^ Patient-specific instrumentation (PSI), computer-assisted navigation, and augmented-reality intraoperative navigation (ARIN) help surgeons enhance surgical precision by reducing malpositioning, optimizing baseplate stability, and potentially preserving bone stock.^2^

Despite technological advances and their proven impact on baseplate fixation metrics, direct comparisons of patient-reported and clinical outcomes across navigation modalities remain limited. Early data suggest that modern navigation systems and conventional techniques yield similar short-term results.^7^^,^^11^^,^^15^ The purpose of this review is to summarize the current literature on glenoid baseplate planning and guidance methods in rTSA. The review will evaluate how each approach influences the accuracy of implant positioning, as well as postoperative function and implant survivorship.

## Preoperative planning methods

### Plain radiographs

Standard radiographic views (anteroposterior, Grashey “true” anteroposterior, scapular Y, and axillary views) historically served as the cornerstone of preoperative evaluation, facilitating assessment of joint-space narrowing, osteophytes, humeral head migration, and glenoid wear.^27^ These images supported freehand guide-pin placement,^12^ albeit with notable limitations. Minor changes in beam angle or patient positioning during radiographic acquisition can significantly distort measurements of glenoid version and inclination.^3^^,^^25^ Nyffeler et al demonstrated that plain radiographs can overestimate glenoid retroversion by up to 21° in 86% of cases compared to computed tomography (CT) scans.^25^ Furthermore, Bischofreiter et al reported poor inter-rater agreement for conventional radiographic templating with values ranging from 33.3% to 83.3%, whereas 3D CT-based planning showed a deviation of only 13.3%.^1^ These shortcomings have led to a shift away from plain radiographs as the standard, paving the way for more reliable cross-sectional imaging techniques, such as 2D and especially 3D CT planning.^26^

### 2D vs. 3D CT planning

While the superiority of 3D CT over 2D imaging is well established, it forms the basis for subsequent innovations in implant positioning and navigation.^13^^,^^26^ Olaiya et al reported that 2D CT scans underestimated glenoid version by up to 5° and inclination by 1.7°, compared to 3D reconstructions.^26^ Furthermore, posterior glenoid bone loss was overlooked in more than half of the cases when relying on 2D images, which jeopardized both baseplate seating and screw placement.^10^ In contrast, dedicated 3D imaging enables virtual templating of screw length, trajectory, and baseplate size with minimal deviation from reality during surgery.^21^^,^^28^

### Intraoperative guidance

Modern intraoperative guidance modalities have been developed to translate precise preoperative plans into exact implant placement: PSI, computer-assisted intraoperative navigation, and ARIN. Each technique has been validated through cadaveric and clinical studies, which have demonstrated incremental improvements in guidewire accuracy, baseplate orientation, screw selection, and placement.

### Patient-specific instrumentation

PSI integrates preoperative 3D templating directly into the surgical workflow by producing custom drill guides that match each patient's unique glenoid anatomy. After finalizing a CT-based plan, 3D-printed guides and corresponding models are manufactured for intraoperative use.^41^ Cadaveric and clinical studies have demonstrated that PSI narrows the gap between planned and achieved component placement.^9^^,^^34^^,^^40^, ^41^, ^42^ For example, Verborgt et al reported good to excellent concordance between planned and actual baseplate and screw positions in 85% of cases, with mean deviations of 4.4° in version, 5.0° in inclination, and 2.4 mm at the entry point, and screw angulation errors of 2.8°-5.3°, demonstrating PSI's ability to accurately reproduce virtual templates.^40^ Similarly, Lee et al found mean differences between planned and actual baseplate version, inclination, and rotation of 2.7°, 0.9°, and 1.0°, respectively, and translation error of 1.7 mm, confirming high reproducibility.^20^ In direct comparison, freehand techniques consistently result in greater deviations from the preoperative plan, with higher rates of outliers and less reliable screw placement. Fleet et al demonstrated that PSI significantly outperformed freehand methods for guide pin inclination and version, with mean errors of 2° and 1° for PSI vs. 5° and 4° for freehand, respectively.^6^ These findings confirm that PSI provides superior accuracy for baseplate and screw positioning compared to freehand techniques in rTSA.

When directly compared to conventional instrumentation, PSI demonstrates significantly improved accuracy and safety. In a clinical study of 39 patients, the mean difference in baseplate rotation between planned and postoperative measurements was 4.5° with PSI vs. 10.6° with conventional instrumentation. The mean differences between planned and actual screw length and angle were also significantly smaller in the PSI group. Notably, screw involvement in the spinoglenoid notch, a surrogate for neurovascular risk, occurred in 10 cases with conventional instrumentation but only 2 cases with PSI (*P* = .014).^19^ Furthermore, a systematic review and meta-analysis found that the number of component outliers was 68.6% with standard instrumentation vs. 15.3% with PSI (*P* = .01), and mean deviations from the preoperative plan for version and inclination were significantly lower with PSI compared to conventional techniques.^41^

### Intraoperative computer-assisted navigation

Intraoperative computer-assisted navigation uses optical trackers affixed to the scapula and surgical instruments to project the surgeon's preoperative plan onto the surgical field in real time.^31^^,^^39^ This allows surgeons to continuously visualize the intended trajectory, select longer screws, and minimize vault breaches.^36^ Verborgt et al found that navigated specimens exhibited tighter error ranges of ±8° for both version and tilt compared to freehand controls with error ranges of ±12° for version and ±16° for tilt.^39^ A direct comparison with conventional instrumentation confirms that navigation improves the precision of baseplate and screw end points and angulation, particularly for the inferior screw and baseplate end point.^38^ It has further been shown that navigation reduces rates of glenoid vault perforation and suprascapular fossa penetration compared to conventional techniques.^15^

### ARIN

ARIN uses a head-mounted display to overlay a holographic 3D glenoid model onto the surgeon's view.^4^^,^^17^^,^^18^^,^^29^^,^^30^ Studies using cadaveric and 3D-printed bone models have confirmed that ARIN reliably directs the initial Kirschner wire,^17^^,^^18^ yielding mean entry-point deviations of just 1.7 ± 0.8 mm and angular errors of 1.6° in version and 1.7° in inclination.^30^ When compared to freehand techniques, ARIN significantly reduces errors in guide pin inclination and version. An in vitro study demonstrated that ARIN and PSI had comparable mean inclination errors (∼2°) and version errors (∼1°), both substantially lower than freehand errors (5° inclination, 4° version), with total global errors also significantly reduced by ARIN and PSI relative to freehand methods. This confirms that ARIN matches PSI in accuracy and outperforms freehand techniques.^6^ Compared to PSI, ARIN offers the advantage of real-time intraoperative feedback without the need for patient-specific guides, potentially improving adaptability during surgery. Cadaveric studies report mean trajectory deviations of 2.7° and entry point deviations of 2.3 mm with ARIN, values consistent with PSI accuracy ranges. The feasibility and reliability of ARIN have been confirmed in multiple studies, supporting its role as a promising alternative to PSI for accurate baseplate fixation in rTSA.^17^^,^^18^ Importantly, ARIN enables even less experienced or younger surgeons to achieve expert-level precision in implant placement, as the real-time holographic overlay and intraoperative feedback compensate for limited tactile or visual experience, though operative times may be increased.^18^ Overall, ARIN provides a reliable and reproducible method for accurate baseplate positioning in reverse shoulder arthroplasty, with particular benefit for less experienced surgeons by narrowing the gap in technical performance compared to experts.

## Comparison of clinical outcomes

### PSI vs. conventional techniques

To date, it was shown that, despite the improved precision of PSI, functional outcomes and patient-reported measures at midterm follow-up are equivalent to those achieved with conventional techniques. In a multicenter trial by Hwang et al, 178 shoulders underwent identical 3D CT templating before randomization to either conventional or PSI-guided glenoid pin placement. At two years, both groups recorded similar American Shoulder and Elbow Surgeons scores, as well as comparable rates of patients reaching the minimal clinically important difference, substantial clinical benefit, and patient-acceptable symptom state. Strength and range of motion assessments revealed only negligible differences, slightly greater strength in the PSI group and improved internal rotation with standard guides, differences attributable to minor lateralization shifts rather than meaningful functional gains. Although the use of PSI led to marginally higher strength values, these variations fell within the expected range of surgical variability and did not translate into clinically meaningful advantages. Moreover, complication rates were low and similar between the PSI and conventional groups and showed no association with the accuracy of baseplate positioning.^12^ Elsheikh et al corroborated these findings in their study with a 2-year follow-up, reporting no significant differences in pain relief, range of motion, or overall satisfaction between PSI and conventional instrumentation; complications in their cohort were rare but instructive, with one patient requiring revision for an excessively long superior baseplate screw and another experiencing glenosphere disengagement from the baseplate during simple abduction six months after a conventional rTSA.^5^

### Intraoperative navigation vs. conventional techniques

Clinical outcomes comparing intraoperative navigation to conventional techniques in rTSA show that intraoperative navigation yields similar or only modestly improved patient-reported outcomes, range of motion, strength, pain, and satisfaction.^7^^,^^11^^,^^35^^,^^43^ Holzgrefe et al found that, at two years, navigation-assisted rTSA had slightly higher absolute forward elevation (135° vs. 129°; *P* = .023), external rotation (39° vs. 32°; *P* = .003), and Constant scores (71.1 vs. 65.5; *P* = .003) compared to conventional methods. However, when improvements from baseline were analyzed, both groups achieved equivalent gains in range of motion and functional scores, suggesting the observed between-group differences may not be clinically meaningful. Patient satisfaction and pain scores were also similar, and complication rates, including scapular notching (3.1% vs. 8.0%) and revision (0.9% vs. 3.5%), were numerically lower with navigation but did not reach statistical significance.^11^ Youderian et al reported that navigation was associated with small but statistically significant improvements in internal rotation, external rotation, maximum lifting weight, and several patient-reported outcome measures. However, pain and satisfaction remained comparable between groups, and there was no difference in revision, loosening, or major complication rates.^43^ Furthermore, Kim et al confirmed that there were no differences in patient-reported outcome and pain scores after 30 months in an Asian cohort, despite better implant tilt and fewer vault breaches with navigation.^15^ Gaj et al also found no significant differences in patient-reported outcomes, range of motion, or satisfaction at 16 months between navigation and conventional groups,^7^ and Troiano et al similarly observed no significant differences in Constant or Disabilities of the Arm, Shoulder, and Hand scores at 41 months.^35^

## Discussion

Accuracy and precision of implant position in rTSA are consistently enhanced by PSI, intraoperative navigation, and ARIN compared to conventional or freehand techniques.^6^^,^^19^ By facilitating accurate baseplate version and inclination and enabling optimally placed, longer screws, these systems may minimize malposition and mechanical outliers, both key drivers of implant failure.^6^^,^^15^^,^^19^^,^^41^

Yet, despite these clear technical advantages, short-term and midterm clinical outcomes such as functional scores, pain relief, or patient satisfaction remain comparable between guided and conventional methods.^7^^,^^11^^,^^35^^,^^43^ Whether the superior radiological accuracy of PSI and navigation will translate into improved long-term implant survival or reduced loosening is still unknown, as most studies lack extended follow-up.

When it comes to complications, guided techniques do offer measurable benefits. PSI markedly reduces spinoglenoid notch breaches, with one study reporting 2 cases vs. 10 in the conventional group (*P* = .014), thereby lowering the risk of neurovascular injury.^19^ Intraoperative navigation has also shown to lower central vault perforations (52.4% down to 17.7%; *P* = .04),^24^ and trends toward fewer scapular notches (3.1% vs. 8.0%) and revisions (0.9% vs. 3.5%), even if these latter differences have yet to reach statistical significance at early follow-up.^11^ Collectively, these improvements in reproducibility and technical safety may, in time, reduce long-term failure rates.

These benefits come with trade-offs. Navigation prolongs operative time,^2^^,^^16^^,^^33^ Kircher et al found an increase from 138 ± 18.4 to 169.5 ± 15.2 minutes (*P* = .001),^16^ largely due to the learning curve and altered workflows of surgeons.^2^ PSI avoids intraoperative delays but requires preoperative manufacturing and logistics, but with emerging reusable instruments mitigating these issues.^23^ Importantly, neither approach has been linked to higher complication rates from longer surgeries.^16^

A notable limitation of PSI, contrasting with intraoperative computer-assisted navigation or ARIN, is the absence of intraoperative verification of the final implant position. With PSI the surgeon must rely on preoperative planning and the guide fit. There is no built-in method to confirm at the end of the procedure that the planned target has been achieved. In contrast, intraoperative computer-assisted navigation and ARIN can provide immediate feedback on the final version, inclination, and screw trajectories, enabling intraoperative adjustments when necessary. This capacity for real-time confirmation may be particularly valuable in complex anatomy or when achieving the planned alignment is uncertain.^6^

Finally, both PSI and navigation level the playing field for less experienced surgeons by standardizing critical steps in implant placement, an advantage in complex cases or low-volume centers.^14^^,^^23^^,^^38^

In summary, while modern guidance tools have not yet demonstrated superior short-term functional outcomes, they do enhance implant positioning accuracy and reduce specific technical complications. Whether these gains will translate into better long-term survivorship awaits confirmation in studies with longer follow-up.

## Conclusion

Although PSI and navigation significantly improve the accuracy of glenoid baseplate positioning in rTSA, there has been no consistent improvement in short-term functional or patient-reported outcomes. However, their potential to reduce complications represents a meaningful clinical benefit that should not be overlooked.

## Disclaimers:

Funding: No funding was disclosed by the authors.

Conflicts of interest: Doruk Akgün serves as a consultant for Medacta International. The other authors, their immediate families, and any research foundations with which they are affiliated have not received any financial payments or other benefits from any commercial entity related to the subject of this article.
